# Enabling access to new WHO essential medicines: the case for nicotine replacement therapies

**DOI:** 10.1186/1744-8603-6-22

**Published:** 2010-11-19

**Authors:** Sandeep P Kishore, Asaf Bitton, Alejandro Cravioto, Derek Yach

**Affiliations:** 1 Weill Cornell/Rockefeller/Sloan-Kettering Tri-Institutional MD-PhD Program, New York, New York, USA; 2 Division of General Medicine at Brigham and Women's Hospital and the Department of Health Care Policy at Harvard Medical School, Boston, Massachusetts, USA; 3 Executive Director of the International Centre for Diarrhoeal Disease Research, (ICDDR, B), Dhaka, Bangladesh; 4 Senior Vice President for Global Health Policy at PepsiCo, Inc, Purchase, New York, USA and is the former Executive Director for the World Health Organization, Geneva, Switzerland; 5 420 E 70th St, Ste 10M, New York, NY 10021

## Abstract

Nicotine replacement therapies (NRT) are powerful tools for the successful treatment of nicotine addiction and tobacco use. The medicines are clinically effective, supported by the Framework Convention on Tobacco Control, and are now World Health Organization-approved essential medicines. Enabling global access to NRT remains a challenge given ongoing confusion and misperceptions about their efficacy, cost-effectiveness, and availability with respect to other tobacco control and public health opportunities. In this commentary, we review existing evidence and guidelines to make the case for global access to NRT highlighting the smoker's right to access treatment to sensibly address nicotine addiction.

## 

Tobacco use kills 5.4 million people annually. Even if no children started smoking in the future, 8.3 million people will die annually of tobacco-related diseases by 2030. Unless tobacco cessation and control vastly improves, the death toll from tobacco this century will easily reach an estimated 1 billion deaths [1]. In this paper, we discuss the benefits and challenges of enabling access to pharmacotherapies [nicotine replacement therapies (NRTs)] to treat nicotine dependence and bolster tobacco cessation in low and middle income countries (LMIC).

In March 2009, NRTs (specifically, nicotine gums and patches) were added to the Model List of Essential Medicines by the World Health Organization (WHO) [2]. Essential medicines are defined as those that satisfy the priority health care needs of the population, and the Essential Medicines List (EML) is used by over 160 governments as a guide for determining which medicines should be made available to their citizens at low cost (http://www.who.int/medicines/en/ and Figure 1). The addition of a medicine to the international EML directly encourages individual nations to add the drug to their national EML and to internal drug registries. This is an important logistical step. Many countries (e.g. South Africa) restrict drug importations to medicines on national EML and registries. Similarly, several foundations and major charities base their medicine supply on the WHO EML. Medicines not on the international or national EML are often not available or are simply unaffordable in LMIC.

**Figure 1:**
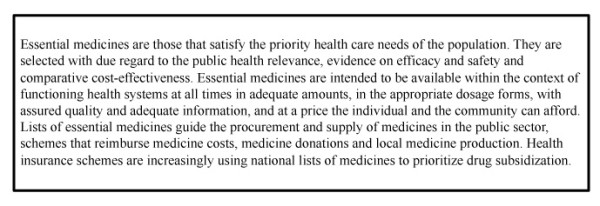
**The concept of essential medicines**.

In this context, we argue that in light of the increasing global burden of tobacco dependence and the clinical utility of NRT, these new WHO essential medicines can and should be available more widely. Let us be clear at the outset. We do not argue that every smoker in a given population should use NRT to quit. Rather, we posit that a nicotine-dependent individual in a low resource country should have the opportunity to affordably access the medicines to treat his or her tobacco dependence.

## Clinical Cessation with NRT: The Evidence

Tobacco dependence treatment "includes (singly or in combination) behavioral and pharmacological interventions such as brief advice and counseling, intensive support, and administration of pharmaceuticals, that contribute to reducing or overcoming tobacco dependence in individuals and in the population as a whole [3]." In this commentary, we focus specifically on access to NRT, one aspect of tobacco dependence treatment. NRT represent a specific class of nicotine-delivering pharmaceuticals that help people stop smoking by curbing nicotine withdrawal symptoms.

The most recent Cochrane review from 2008, using pooled data from over 40,000 people in 111 trials between 1979-2007, shows that the relative risk of sustained tobacco abstinence with NRT at 6 months or more was 1.58 compared with controls (95% Confidence Interval (CI): 1.50-1.66) [4]. According to the 2008 US Public Health Service (USPHS) guidelines, the number of smokers needed to be treated with NRT to achieve one quit ranged from 8 for long term use of (> 14 weeks) nicotine gum to 10 for nicotine patch and 20 for short term use of nicotine gum [3]. The estimated absolute 6-month smoking abstinence rates for NRT patch and long term gum were 23.4% and 26.1%, respectively, compared to 13.8% for placebo [3]. Given the efficacy and relatively low cost of NRT, the addition of NRT to the EML represents an important potential milestone for furthering the goals of global tobacco control while moving towards equitable access to essential medicines.

NRT and brief counseling (in the clinical setting or through telephone quitlines) represent effective and inexpensive clinical treatment approaches for helping the large number of individuals dependent on nicotine worldwide to quit using tobacco. Group and individual counseling increases significantly the odds of successful quitting by a factor of 1.3-1.7 above baseline [3]. The combination of counseling with medications works to synergistically increase quit rates by a factor of 1.7 over counseling alone [3]. We argue that in concert with effective societal approaches to reduce tobacco use through higher tobacco taxes and social denormalization of tobacco use ("unassisted cessation"), clinical treatment options such as counseling and NRT should be available to help those that cannot quit without support. To only promote unassisted cessation without making available NRT to those who cannot or will not quit on their own denies the known addictive nature of nicotine, as well as the known harm of exposing nicotine addicts to stigmatizing policies and economic burdens without the option to help them quit.

**Figure 2:**
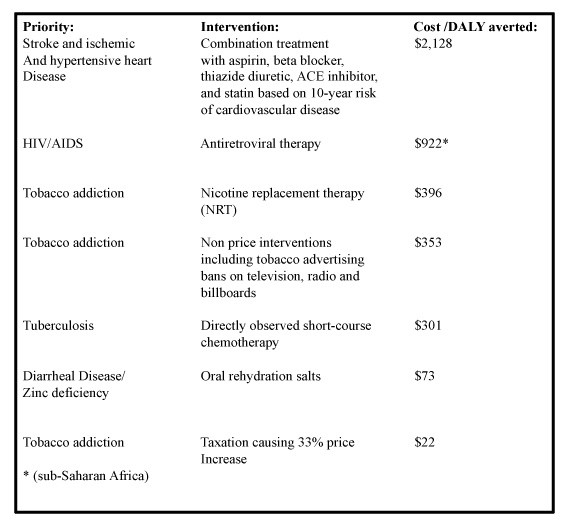
**Cost-effectiveness (Cost (USD) per DALY averted) of selected health priorities in LMICs, 2006 (adapted from **[28].

By present international standards, NRT can be considered a cost-effective clinical intervention-one estimate predicted that achievement of 25% global coverage with NRT would cost US$ 276-279 per disability-adjusted life year averted in East Asia [5]. In a low-income country, the cost of cessation therapy with short-term use of NRT (e.g. 8 weeks of patches) is US $50 [5]. Prices of NRT, while still relatively high in the developing world, may be falling. In India, the price of NRT as of 2003 was approximated at $0.65 per day for a three month course in 2003 - still high, but 12% of that in the United States[5]. In the widely respected Disease Control Priorities Project published in 2006, Prabhat Jha and colleagues estimated that providing NRT with an effectiveness of even 1% above baseline in low and middle countries could save nearly 3 million lives over the next half century; if the effectiveness is 5%, they estimated that over 14 million lives would be saved [6]. These estimates translate into a reasonable range of cost effectiveness in low and middle income countries; comparable to other proven public health strategies (See Figure 2).

Perhaps the most convincing evidence comes from the real-word distribution campaigns of NRT. In 2003, the New York City Department of Health and Mental Hygiene, leaders in instituting public smoking bans, began a widely publicized campaign to distribute free NRT [7]. In total, they estimated that they sent nicotine patches to roughly 5% of the total adult smoking population in New York City during the short campaign [7]. All of the smokers smoked at least 10 cigarettes daily and 60% of the smokers were non-white, foreign born or from low-income neighborhoods. When checked 6 months later, more NRT recipients than the control group members successfully quit smoking (33% vs 6%, p = 0.0001); with over 6000 successful quits, this program yielded a cost of $464 per quit [7].

Recent data specific to LMICs suggest that the medicines would be as efficacious as they are in developed countries, provided the delivery infrastructure is present. These include a study of 341 patients in Brazil that showed greater than 25% 12-month cessation rate for those receiving counseling and NRT vs. 14.5% for counseling alone [8], along with other studies in Hong Kong [9] and Venezuela [10] showing similar efficacy. However, the effectiveness of NRT in the real-world in LMICs remains untested.

## The Framework Convention on Tobacco Control and Tobacco Treatment

In light of the tremendous toll of tobacco use, the world's first global public health treaty, the Framework Convention on Tobacco Control (FCTC), was developed and first signed in 2003 by WHO member states http://www.who.int/fctc/en/. The FCTC emphasizes both a reduction in the demand and supply of tobacco through the implementation of the MPOWER package of policies [1]. These policies include Monitoring tobacco use, Protecting people from secondhand smoke, Offering treatment for tobacco dependence, Warning people of the dangers of tobacco use, Enforcing advertising bans, and Raising tobacco taxes [1].

To date, however, the majority of global efforts have focused on all the items of the MPOWER package except offering treatment. Some have even remarked, only in half-jest, that the "O" stands for "orphan," not offer treatment. However, Article 14 of the FCTC clearly requires the 169 signatory nations:

"to develop and disseminate appropriate, comprehensive and integrated guidelines based on scientific evidence and best practices, taking into account national circumstances and priorities...and take effective measures to promote cessation of tobacco use and adequate treatment for tobacco dependence...[and] to collaborate with other parties to facilitate accessibility and affordability for treatment of tobacco dependence including pharmaceutical products..."[11].

Progress on implementing the FCTC protocols remains mixed. In 2008, comprehensive treatment services to help users overcome tobacco dependence were available in only 17 countries representing 8% of the world's population; none of these countries were in the developing world [12]. At the 14th World Conference on Tobacco or Health in Mumbai, India in March 2009, the convened body recommended that by 2012, the majority of parties to the FCTC should begin to provide national tobacco cessation efforts as recommended under Article 14.

While the FCTC is catalyzing some tobacco control success worldwide, the tobacco industry continues to market aggressively and successfully. Smoking prevalence remains close to 25% in the 30 high- and middle-income countries belonging to the Organisation for Economic Cooperation and Development (OECD) and is increasing in LMIC, especially among women. Currently, in Matlab, Bangladesh, nearly 70% of men and 33% of women smoke or use smokeless tobacco [13]. The effect of these practices is striking with 79% of deaths in Matlab attributed at present to non-communicable diseases, many of which are linked to the use of tobacco [14]. Male Bangladeshi smokers currently spend twice as much on tobacco as on education, health, and other household expenditures combined [15]. To this end, the inclusion of NRT on the EML may help catalyze the inclusion of tobacco treatment into clinical systems across the developing world.

### Challenges & Counter-arguments

#### Alternative Cessation Methods

Are we in danger of over-hyping NRT for use in developing countries or "over-medicalizing" the treatment of tobacco use? We acknowledge that these medicines are not a panacea, and that most evidence for their efficacy comes from the developed world. NRT and behavioral programs typically help fewer than 30% of smokers who use them to quit over the long-term. Cultural norms around tobacco use and cessation, as well as knowledge about the harms of tobacco, are different in LMIC compared to developed countries. Investment in upstream approaches to modify the social climate around tobacco use, bans on advertising and promotion, and clean indoor air laws are important first approaches in resource-scarce public health environments[16]. While there is some controversy around the population-wide or "real-world" effect of NRT in developed countries [17,18], new evidence from Massachusetts suggests a likely association between increased insurance coverage for, and use of, smoking cessation medications with increased quit attempts and decreased smoking prevalence among low-income smokers on public insurance [19]. Furthermore, Grassi et al (2009) showed that concomitant clean indoor air laws can synergistically increase rates of successful cessation among smokers treated for tobacco use in a clinical setting[20].

What is clear, regardless of one's point of view on the best method of cessation, is that nicotine-dependent patients deserve the right to affordable access for these treatments if they so choose. This is especially true considering the cost-effectiveness of NRT compared to many other widely used clinical interventions (See Figure 2). For example, combination use of statins and anti-hypertensives, all WHO essential medicines [2,21] used for the control of cardiovascular disease is much less cost-effective than NRT. Further, the use of directly observed chemotherapy for tuberculosis or tobacco advertising bans feature comparable cost-effectiveness to NRT use - estimates that hold up in both developed and developing world settings.

To this end, the newly acquired WHO essential medicine status can and should encourage WHO member states in low and middle income regions to register NRT for use in their countries. While more evidence is needed to understand quit rates using NRT in LMICs as well as optimal ways to provide treatment services within resource-poor settings, there is no *prima facie *reason to believe that NRT is not effective in the developing world (reviewed below). In fact, the EML committee did not demand confirmatory proof of effectiveness in the developing world before including other medications such as proton pump inhibitors and oncologic chemotherapeutics in the EML.

Just how NRT use is best parceled out within the larger tobacco treatment clinical context in low resource settings remains an open question deserving of further rigorous empirical study. To be sure, the preventive and treatment approaches need not be viewed as mutually exclusive, even in resource-starved environments. The approaches can complement one another [20] and smoking cessation services are most effective in the context of coordinated tobacco control [12].

#### Role of the Pharmaceutical Industry

In a field sensitive to conflict of interest and accustomed to dealing with now infamous tactics of tobacco industry deceit [22] there is some understandable skepticism of the role of pharmacotherapy in cessation [23], particularly regarding the reliability of industry-backed trials or excessive hype over these medicines. This skepticism, however, does not change the fact that the drugs have been shown to work empirically and, based on this evidence, have been incorporated into major national and international health platforms (including the FCTC and EML). The large scale distribution of free NRT by large urban public health agencies underscores this trend [7].

If anything, the essential medicine status of NRT should now help break down structural and price barriers maintained by the pharmaceutical industry that prohibit patients, particularly in lower income countries, from accessing these medicines in an affordable manner (such as being sold in single use quantities). Targeted expansion of generic production of these medicines, including the use of humanitarian licensing and enhanced drug donation schemes to augment NRT availability and drive costs down, are needed urgently for these cessation programs to work in concert with other public tobacco control strategies.

#### Uptake and Delivery of NRT in LMIC

It is unclear whether the packaging and delivery of a pharmacotherapy is tenable currently for many smokers in LMIC. There is little to no cultural awareness of how these medicinal products could help in cessation, particularly in an environment where cessation is not normative. In fact, the use of NRT has been largely untested in LMIC outside of a few studies [5,24]. Task-shifting treatment services, as levered by the HIV/AIDS and Tuberculosis (TB) treatment communities, as well as deregulation of the medicines for over-the-counter treatment can help, but require further detailed study in LMIC for NRT. Cessation efforts should be naturally integrated into ongoing efforts to strengthen primary care. Critically, the process of working with existing treatment providers in LMIC to identity target populations (such as patients with TB) to offer effective counseling and deliver NRT to a larger group of tobacco users worldwide is a necessary step to reduce tobacco use [5,24].

#### Financing

A final thorny issue, currently unanswered, is who will actually pay for the treatments? Is it the role of the individual patient or of the government or even of large pharmaceutical companies distributing these medications for free or at reduced prices? Lists of essential medicines guide the procurement and supply of medicines in the public sector, with several LMIC with social health insurance programs subsidizing coverage of EML-listed medicines. The use of government tax on tobacco revenues could help finance telephone quit lines and clinical cessation services, including distribution of NRT to nicotine dependent patients [9].

It is clear that governments need to prioritize their funding of drugs on the EML proportional to the expected public health gains. Further, WHO and governments must negotiate with NRT manufacturers to ensure that NRTs are affordable in key LMIC markets. Given the growing burden of tobacco, even low-income countries (e.g. Bangladesh) may find ways to prioritize multimodal tobacco control strategies. These strategies, including cessation treatment, can naturally synchronize with a growing political, social and technical movement to drive resources to control non-communicable chronic diseases (NCDs) and their risk factors (particularly tobacco) through a planned United Nations high-level meeting on NCDs in September 2011 [25-27].

As outlined in the most recent WHO MPOWER report on global tobacco, "in the vast majority of low- and middle-income countries, the cost of cessation assistanceis not covered by the government, and 8% of middle income and 29% of low-incomecountries provide no assistance at all." [9] Hence, it is imperative for countries to make the medicines available and more affordable (through drug registration and direct import) to individual nicotine-dependent patients who want them at pharmacies in both urban and rural locales.

Further, it is reasonable for countries to consider stocking NRT for use in ambulatory clinical settings among particularly high-risk tobacco users such as newly diagnosed TB patients or patients with severe chronic obstructive pulmonary disease. Whether a population-based effort to promote more widespread NRT use would be clinically appropriate or economically feasible in the near future remains an open question in need of more study.

## Concluding thoughts

Though a vital part of tobacco control, clinical tobacco treatment is often neglected. It is now clear that neither public health interventions like smoking bans or increased taxes, nor clinical strategies like offering NRT, are sufficient alone. Lasting progress toward reducing the burden of tobacco will only be made by integrating these crucial legislative, financial, and clinical approaches. Coupled with the FCTC and heightened political attention on NCDs and tobacco control, NRT provides another valuable tool for reducing the global burden of tobacco use. It is now more important than ever that countries swiftly register these medicines, and liaise with appropriate generic manufacturers to augment availability and harmonize clinical cessation into systems-level clinical care. The real question now is when will we act?

## Competing interests

DY is a member of the Board of Directors of Vitality USA, a Discovery SA institution that supports tobacco cessation and other preventive measures and is not funded by the pharmaceutical industry or manufacturers of NRT. As a WHO staff member, DY actively pushed for tobacco cessation as part of the WHO Framework Convention of Tobacco Control. SPK is a member of the Board of Directors of Universities Allied for Essential Medicines, a non-governmental organization supporting access to essential drugs and vaccines in resource-poor countries.

## Authors' contributions

All authors contributed to the writing and reviewing of this work.
